# Bibliometric analysis and visualization of Connexin 43 in the field of solid tumor research(2000-2024)

**DOI:** 10.3389/fimmu.2025.1588828

**Published:** 2025-05-09

**Authors:** Caiqi Huang, Xiaoman Liu, Yanhui Feng, Zhesi Xiao, Zhengjia Lu, Lu Wang, Jia Ming

**Affiliations:** Department of Breast and Thyroid Surgery, The Second Affiliated Hospital of Chongqing Medical University, Chongqing, China

**Keywords:** Cx43, solid tumor, bibliometric analysis, phosphorylation, prognostic signature, immunity, resistance

## Abstract

**Background:**

Connexin 43 (Cx43) plays a pivotal role in tumor growth, metastasis, and disease progression. This study employs bibliometric analysis to identify key research trends and emerging hotspots in Cx43-related solid tumor research.

**Methods:**

In December 2024, the Web of Science Core Collection (WoSCC) database was searched for publications on Cx43 in solid tumor research from 2000 to 2024. Bibliometric analysis and data visualization were primarily conducted using CiteSpace, VOSviewer, and Bibliometrix, with a focus on visualizing aspects such as countries, institutions, journals, authors, references, and keywords in the field.

**Results:**

A total of 1,666 publications were retrieved, with the annual number of articles and citations continuing to grow. The United States and China had the highest number of publications, while the University of Western Ontario in Canada was the leading institution, with the most publications by Christian C.G. Nau. Lampe, P.D. was the most cited author. The International Journal of Molecular Sciences was the most frequently published journal, and the Journal of Biological Chemistry was the most frequently co-cited journal. High-frequency keywords included phosphorylation, breast cancer, gastric cancer, prognostic markers, anti-tumor immune response, and drug resistance.

**Conclusion:**

Contemporary research focuses on the role of Cx43 phosphorylation in tumorigenesis, progression, and metastasis, its potential as a prognostic biomarker, and its critical role as an immunotherapeutic target and in tumor drug resistance. These studies provide a comprehensive analysis for a deeper understanding of the role of Cx43 in solid tumors and help to promote further research in this area.

## 1 Introduction

Cancer is a significant public health and economic issue in the 21st century. According to estimates from the International Agency for Research on Cancer (IARC), nearly 20 million new cancer cases and 9.7 million cancer-related deaths occurred globally in 2022. It is projected that by 2050, the number of new cancer cases will reach 35 million. The heterogeneity of the microenvironment in solid tumors presents a major challenge for medical practice (1). There is growing evidence that solid tumors may contain a hierarchy of cancer cells within the normal tissues where malignant tumors initially develop (2). Many studies suggest the existence of a subpopulation of cancer cells, known as tumor-initiating cells, which affect membrane permeability and intercellular communication in normal cells (3). Connexins are the primary protein components of gap junctions and are widely expressed in various human tissues and organs as transmembrane proteins. These proteins play important roles in maintaining normal physiological functions and have been linked to the development of numerous diseases. To date, 21 connexin isoforms have been identified in humans, each named according to its molecular weight. Cx43, a protein encoded by the GJA1 gene, is the most well-studied member of the connexin family and is most closely associated with tumors (4–7).

**Figure 1:**
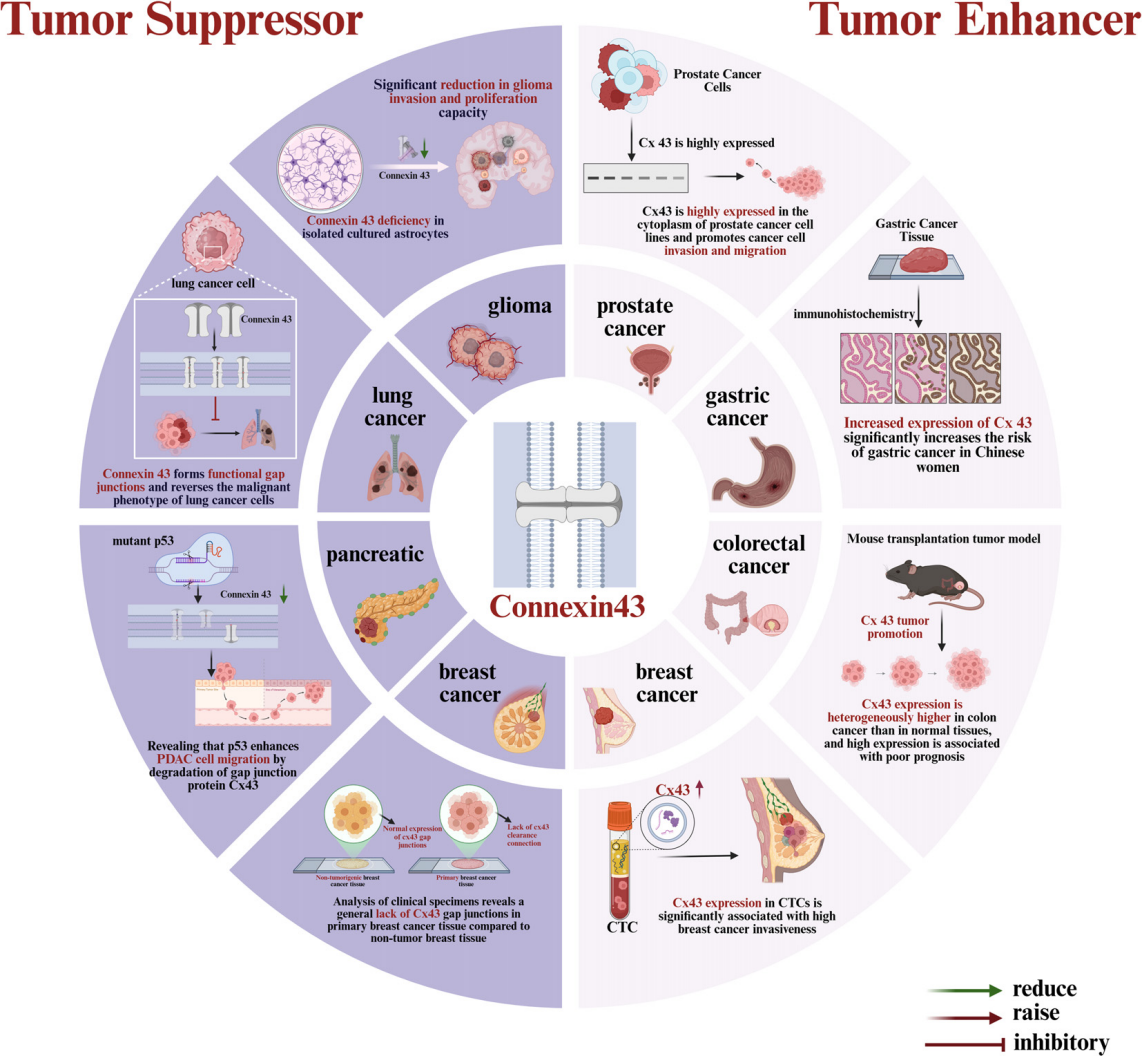
Dual role of Cx43 as a tumor suppressor and enhancer in multiple cancers.

Recent studies have shown that Cx43 plays a yet undefined role in tumorigenesis and development, functioning both as a tumor suppressor to inhibit tumor proliferation and as a tumor enhancer to promote tumor cell migration and invasion (8). This seemingly contradictory role may arise from various factors, including tumor type, cellular localization (membrane versus cytoplasmic localization), post-translational modifications, and interactions with other signaling pathways. The role of Cx43 in different solid tumors is summarized in **Figure 1**. In malignant tumors such as gliomas (9), lung cancer (10), pancreatic cancer (11), and breast cancer (12, 13), Cx43 can act as a tumor suppressor by promoting electrical and chemical signaling between normal cells through the formation of gap junctions, maintaining normal cellular function and inhibiting tumorigenesis (8). Specifically, Cx43 influences intercellular communication via Gap junctional intercellular communication(GJIC) (14) and transmits oncogenic signaling molecules, thus maintaining tissue homeostasis and inhibiting tumor proliferation. For instance, in gliomas, Cx43 expression is associated with low tumor proliferation and a better prognosis (15). However, in tumors such as prostate, gastric, and colorectal cancers, the deletion or aberrant expression of Cx43 may promote tumor cell proliferation and invasion by disrupting GJIC function and interfering with tumor cell communication (16, 17). For example, the deletion of Cx43 causes tumor cells to lose control of their surroundings, enhancing their migration and invasion (18). Furthermore, aberrant expression of Cx43 may promote tumor cell proliferation and anti-apoptotic functions by activating signaling pathways such as MAPK (19). Catalina Asencio et al. demonstrated through immunofluorescence analysis that Cx43 was highly expressed in the cytoplasm of prostate cancer cell lines, promoting cancer cell migration and invasion (20). A clinical study by Yuan et al. found that Cx43 significantly increased the risk of gastric cancer in Chinese women, suggesting it may serve as a potential clinical marker for evaluating the risk of gastric cancer in females (21). Han et al. demonstrated that the lack of Cx43 expression in colorectal cancer was positively associated with cancer metastasis and poor prognosis (22). Additionally, a previous study by our group found that Cx43 expression in circulating tumor cells (CTCs) of breast cancer patients has a pro-cancer effect, reflecting its special role in bloodstream metastasis (23). In summary, Cx43 plays a complex dual role in tumors, and its specific function may vary depending on the tumor type and microenvironment. With the ongoing study of Cx43, growing evidence suggests that it may exhibit different roles at various stages of cancer. Therefore, exploring the role of Cx43 in solid tumors and its potential mechanisms will not only help reveal the molecular basis of tumorigenesis and development but may also provide new targets for tumor diagnosis, treatment, and prognostic assessment.

Bibliometrics evaluates the output and impact of academic research by analyzing the generation and dissemination of literature through quantitative and statistical methods (24), Despite extensive research on Cx43 in solid tumors, there has been a lack of systematic bibliometric analyses to identify research trends and hotspots. To address this gap, this study analyzed the annual publication output, country, institution, author, and journal distribution of Cx43 research in solid tumors based on the Web of Science database from 2000 to 2024. It also constructed collaborative networks and knowledge graphs and identified research hotspots through keyword co-occurrence and citation burst analysis. The study aims to reveal the development background, trends, and research gaps surrounding Cx43 in solid tumors, providing a foundation for subsequent in-depth exploration (25).

## 2 Materials and methods

### 2.1 Search strategy and data collection

**Figure 2:**
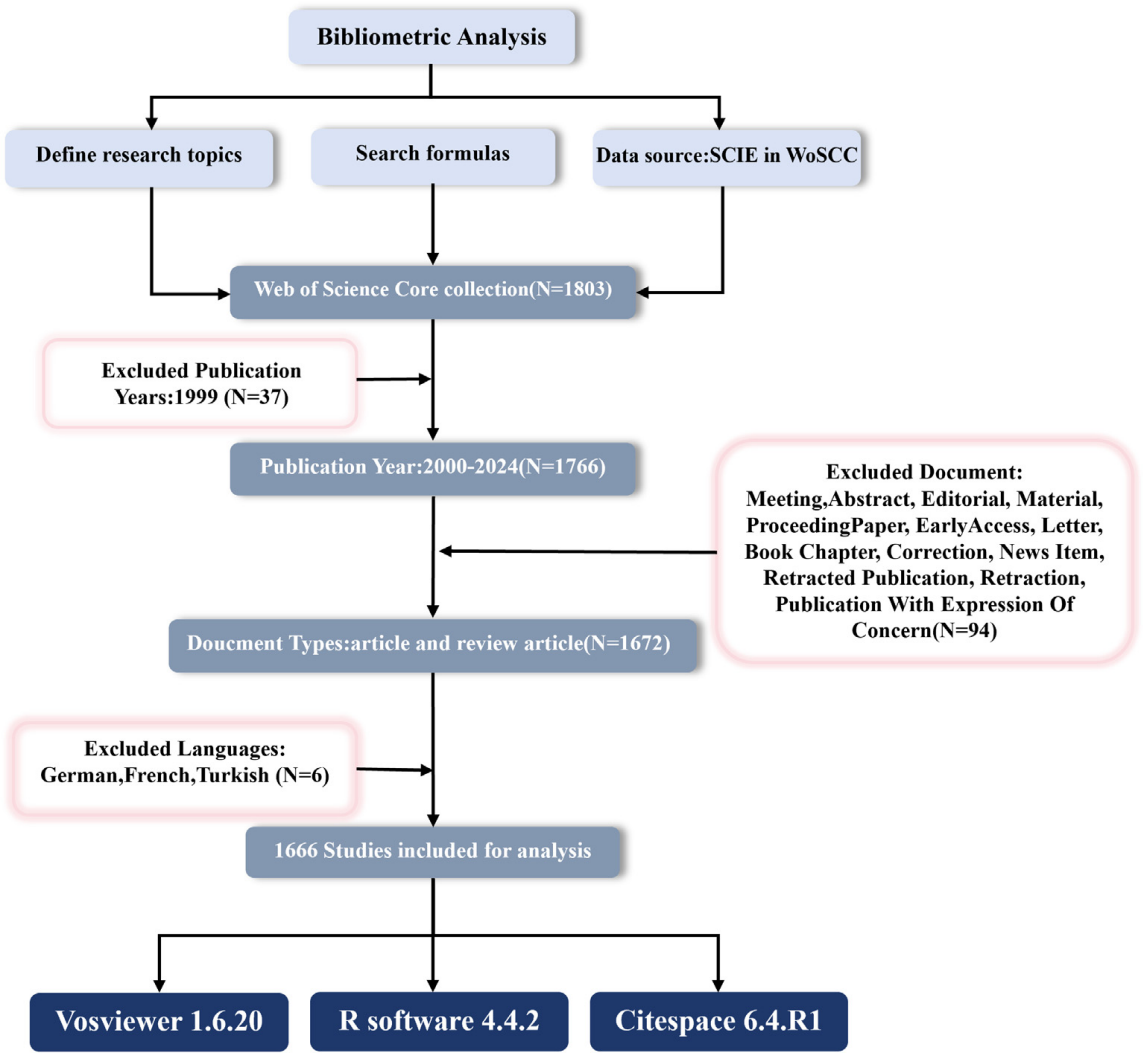
Flowchart of scientometric analysis process Cx43 in solid tumor study.

Web of Science (WoS) is a globally recognized and essential literature database that provides detailed citation information and is widely used for scientific evaluation and academic research (26–28). All searches and data downloads were completed on December 6, 2024, to avoid any bias from database updates. We selected the SCI-EXPANDED index from the WoS core collection and searched for literature on “Cx43” and “solid tumors” from January 1, 2000, to December 6, 2024, using the following search strategy: (TS=(Cx43 OR “Connexin43” OR “Connexin-43” OR “GJA1”)) AND TS=(cancer* OR carcinoma* OR neoplasms* OR tumor* OR tumour* OR malignan*). “TS” denotes the subject, and “*” represents zero or more characters. The initial search yielded 1,766 publications, and after excluding 94 non-articles or reviews and 6 non-English publications, 1,666 publications (1,536 articles and 130 reviews) were included. All documents were downloaded in the “Full Record and Cited References” format, and the process of data extraction, cleaning, and standardization is shown in **Figure 2**.

### 2.2 Data analysis

Using VOSviewer 1.6.20, CiteSpace 6.4.R1, and Bibliometrix for bibliometric analysis and the creation of knowledge graphs, this study leveraged several software tools to analyze and visualize the data. VOSviewer, developed by Eck and Waltman, is a tool designed to build and view bibliometric maps that help quickly identify core literature and research hotspots within a given field (29). CiteSpace, developed by Prof. Chao-Mei Chen, visually maps highly cited and critical literature, highlighting the evolution and research frontiers within an academic field (30). Bibliometrix is an R-based software package (https://bibliometric.com/) primarily used in bibliometrics to provide statistical analysis techniques, as well as a network construction and visualization toolkit (31). In this study, we used VOSviewer to extract and visualize information about authors, co-cited authors, countries, institutions, journals, co-cited journals, and co-cited references. We also built collaborative networks of countries, institutions, and authors. Journal biplot overlays, reference timeline views, keyword co-occurrence clustering, and bursts were analyzed using CiteSpace (26). Keywords were clustered and co-occurred after the data was cleaned by merging synonyms. The Biblioshiny software from the Bibliometrix package was used to create collaborative network maps for each country. Pajek software (version 5.19) was used to adjust the clusters for clarity and aesthetic appeal in the images. Microsoft Excel 2010 was used to illustrate the annual distribution of publications and to demonstrate the publication output and citation trends of Cx43 in solid tumors from 2000 to 2024.

## 3 Results

### 3.1 Annual number of publications, citations and trends

**Figure 3:**
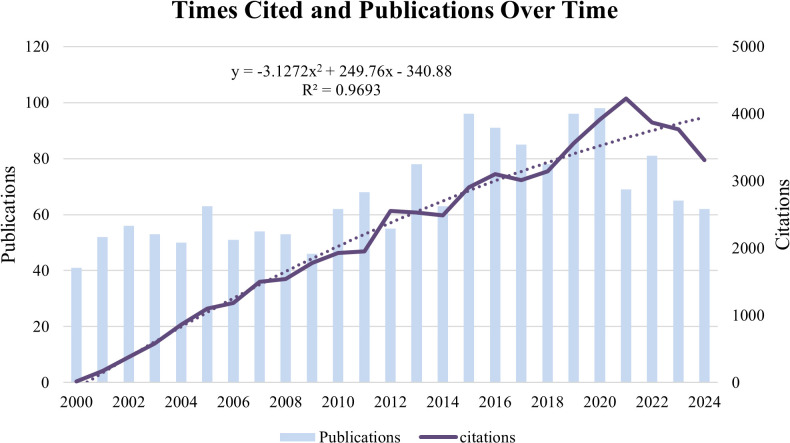
Trends in the annual publications and citations of articles on Cx43 in solid tumor between 2000 and 2024.

**Figure 3** shows the trends in annual publications, citations, and related data for Cx43 in solid tumors. The number of publications from 2000 to 2020 demonstrates a consistent upward trend, with slight fluctuations from year to year. The volume of annual publications was relatively low in the early years (2000–2009) but gradually increased, particularly after 2014, when a more significant rise was observed. The number of publications peaked at 98 in 2020, followed by a slight decline in publications from 2021 to 2024. The number of citations grew from 18 in 2000 to 4,224 in 2021. Using binomial fitting analysis, this study quantified the change in the citation frequency trend for Cx43-related literature. The model effectively identifies key inflection points, cyclical fluctuations, and potential evolutionary directions in the field. The results indicate a high goodness-of-fit (R² = 0.9693), which is close to the theoretical maximum of 1, suggesting that the model is highly effective in explaining the variations in citation data. The negative quadratic term coefficient of -3.1272 reflects a gradual slowdown in the growth of citations.

### 3.2 Country and institutional analysis

Between 2000 and 2024, a total of 61 countries around the world conducted research related to Cx43 and solid tumors. In terms of the number of publications, the United States ranked first with 454 publications, followed by China (433) and Japan (135) in second and third place, respectively. Other countries with more than 100 publications include Canada (134), Germany (134), and France (114). In addition to the number of publications, the number of citations is also an important factor in assessing a country’s influence in the field. The United States had the highest number of citations, totaling 22,275, followed by China (7,800), Canada (6,823), France (5,075), and Germany (4,903). Furthermore, for the top 10 countries by publication volume, we conducted a citation-weighted productivity analysis, an indicator that measures the productivity and impact of a country’s research over a specific time period. The results show that Italy had the highest citation-weighted productivity (n=53.55), followed by Canada (n=50.92), the United States (n=49.06), France (n=44.52), and Spain (n=41.24). Notably, despite being the second-largest country in terms of publication volume, China ranks only 10th in citation-weighted productivity, as shown in **Table 1**.

**Table 1:** Top 10 country with the highest publication outputs investigating Cx43 in solid tumor from 2000 to 2024.

Rank	Country	Documents	Citations	Citations-weighted productivity	Total Link Strength
1	United States	454	22275	49.06	248
2	China	433	7800	18.01	72
3	Japan	135	3712	27.50	73
4	Canada	134	6823	50.92	87
5	Germany	134	4903	36.59	81
6	France	114	5075	44.52	89
7	Italy	71	3802	53.55	42
8	South Korea	60	1726	28.77	19
9	United Kingdom	51	1742	34.16	59
10	Spain	45	1856	41.24	33

**Figure 4:**
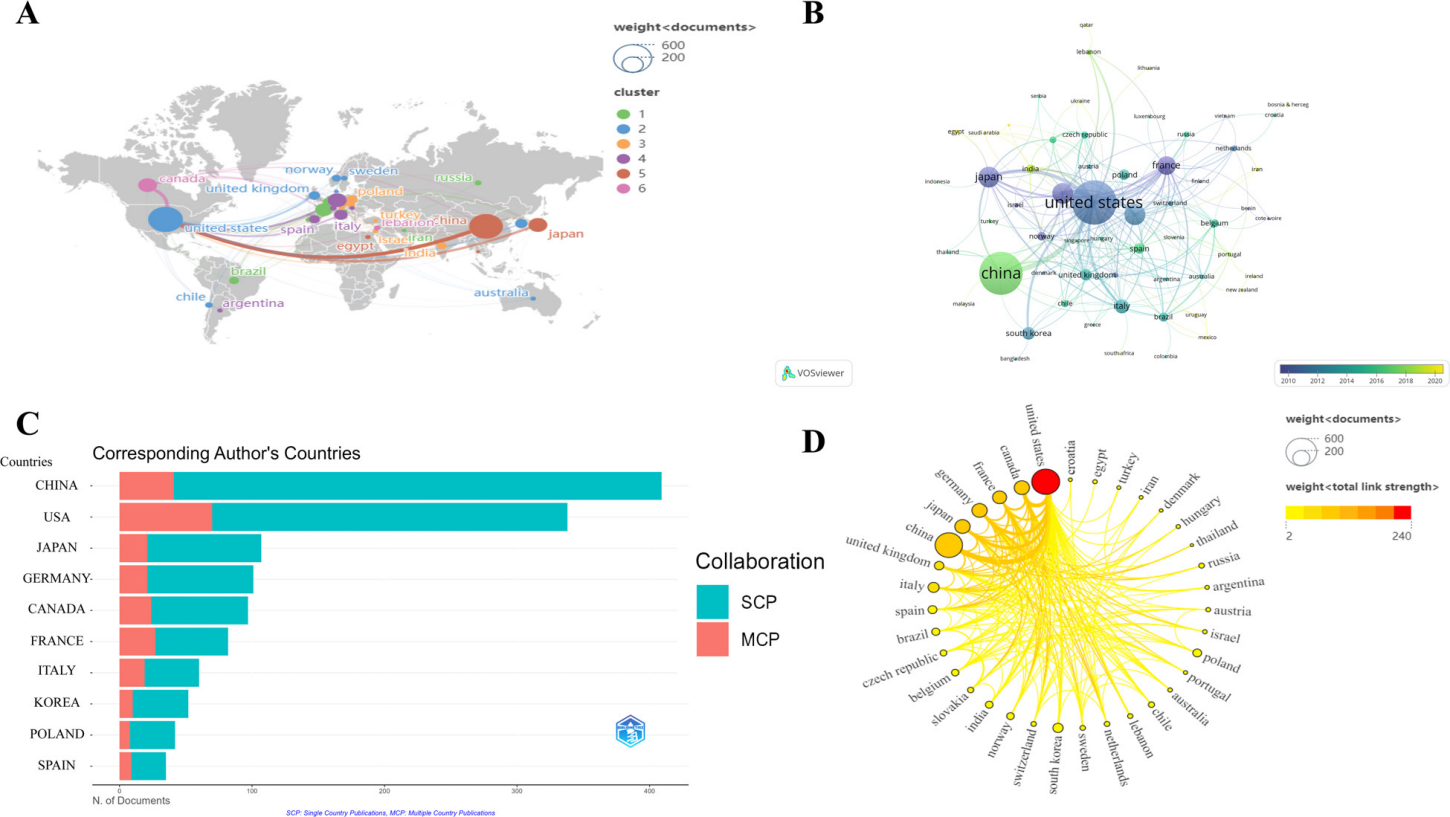
Distribution of countries in Cx43 and solid tumor. **(A)** Geographic visualization map of Countries/regions collaboration. **(B)** Overlay map of countries’ average publication year. **(C)** Countries and the number of publications according to the country of corresponding authors (Top 10). **(D)** The number of national publications and the intensity of cooperation.

The global academic collaboration network among major countries is shown in **Figure 4A**, which illustrates the relationships among 61 countries. Each circle represents a unique country, with the size of each circle corresponding to the number of articles published in that country or region. The connecting lines represent collaborative relationships between countries. The United States and China are the largest nodes in the graph, indicating their high influence and intense collaboration in this field, as well as their roles as core nodes. The connecting lines between European countries are also quite dense, especially among Germany, Italy, and Sweden, reflecting the strong regional cooperation in this field. In **Figure 4B**, the color of each node represents the average publication year of the country, with the color fading from purple to yellow, indicating a timeline from earlier to more recent publications. The color distribution allows us to observe the trend in the distribution of scientific research output over time in different countries. Countries with yellower nodes, such as China, India, Malaysia, and Egypt, indicate more recent scientific output in the field of Cx43 and solid tumors. In contrast, countries with nodes closer to purple, such as the United States, Japan, and France, show that their research output has been more concentrated in earlier years. **Figure 4C** shows the distribution of the nationality of corresponding authors and the degree of international collaboration. MCP (Multi-Country Publication) indicates the number of papers co-authored with foreign researchers, while SCP (Single Country Publication) indicates the number of papers co-authored domestically. In terms of publication volume, the average MCP ratio for the top 10 countries is 22.43%, suggesting that their research collaborations are primarily domestic. China, Poland, and Korea have lower levels of international collaboration, with MCP ratios of 10%, 19%, and 19.2%, respectively. This difference may be due to factors such as geographical location, cultural background, and language barriers. In contrast, countries such as France, Italy, and Spain primarily disseminate research through international cooperation, with MCP rates of 32.9%, 31.7%, and 25.7%, respectively. **Figure 4D** displays the volume of national publications and the degree of international cooperation. The United States has the highest number of publications and the most extensive international cooperation. Notably, China has the second-highest number of publications, but its degree of international collaboration is not as high as that of the United States. Canada, while having a smaller number of publications, ranks third in international cooperation. Overall, this figure illustrates the network of academic collaboration between countries, highlighting the core countries’ positions and their extensive collaborative relationships.

**Table 2** shows the top 10 institutions in terms of the number of publications from 2000 to 2024. The University of Western Ontario ranks first with 40 publications, the highest number of citations (n=2,343), and a total link strength of 32. The University of British Columbia ranks second with 39 publications, 2,015 citations, and the highest link strength (n=34), indicating its strong collaboration with other institutions. The top two institutions are from Canada, highlighting the scientific activity of Canadian research on Cx43 in the field of solid tumors and its academic influence. Michigan State University performs well in terms of citations (n=1,399), although it has a slightly lower number of publications (n=31). China Medical University is ranked fifth. Among these top 10 institutions, three are from China, indicating that China has begun to make an impact in the field. However, academic influence and research collaboration remain limited. In the future, China could further expand its international cooperation to strengthen its position in global scientific research.

**Table 2:** Top 10 organizations with the highest publication outputs investigating Cx43 in solid tumor from 2000 to 2024.

Rank	Organization	Publications	Citations	Total Link Strength
1	Univ Western Ontario	40	2343	32
2	Univ British Columbia	39	2015	34
3	Seoul Natl Univ	34	1171	18
4	Michigan State Univ	31	1399	23
5	China Med Univ	26	610	34
6	Sun Yat Sen Univ	25	517	17
7	Univ Poitiers	24	694	32
8	Fudan Univ	23	435	10
9	Jagiellonian Univ	20	426	0
10	Mcgill Univ	20	1275	20

### 3.3 Visualization and analysis of authors and co-cited authors

**Figure 5:**
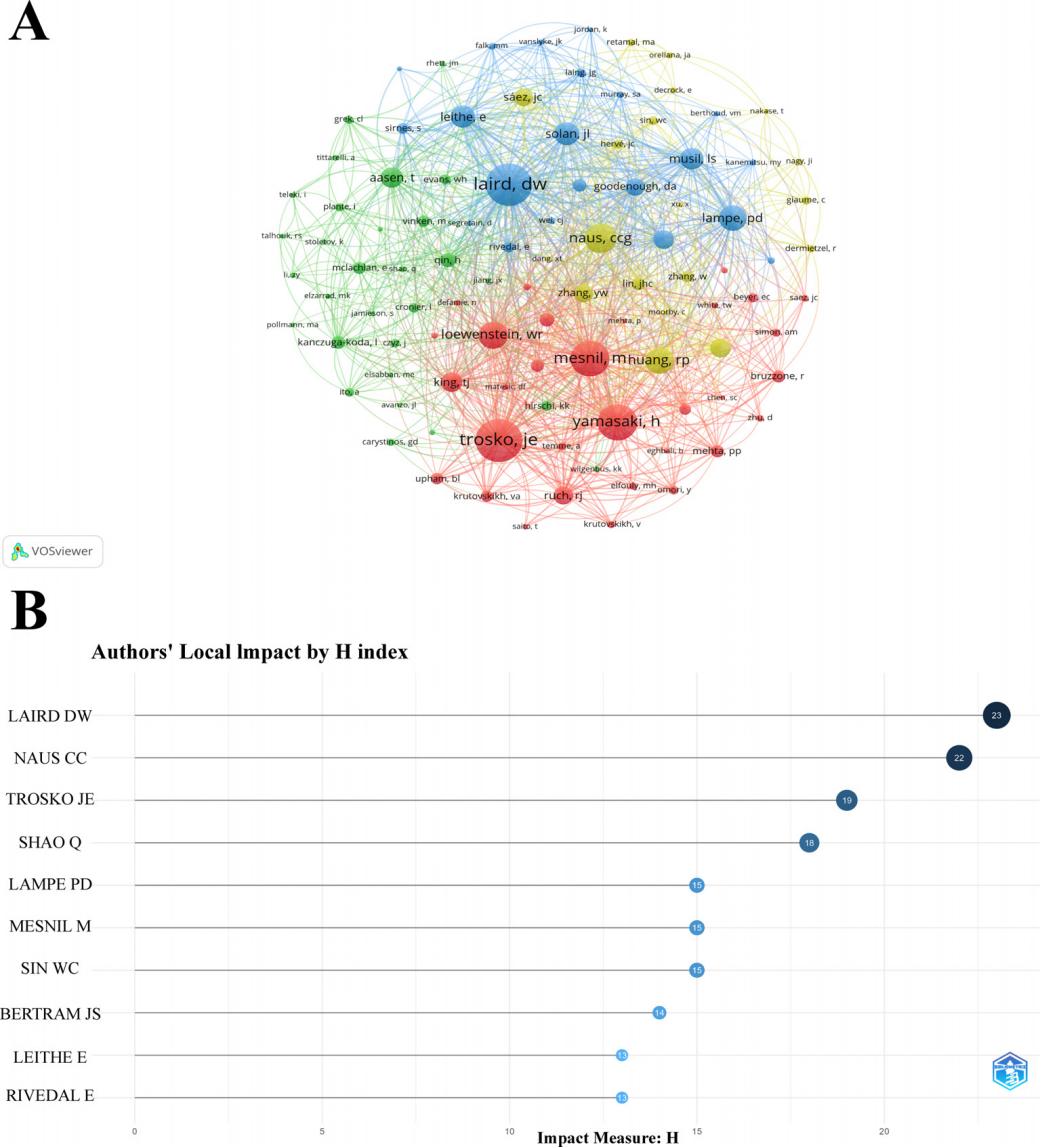
**(A)** Network visualization map of the top 100 co-cited authors with the highest number of citations. **(B)** Author local impact according to the H index.

Based on the analysis of the retrieved journals, an author and co-cited author analysis was performed, involving a total of 8,563 authors and 38,433 co-cited authors in the study of Cx43 in solid tumors. **Figure 5A** presents a co-authorship network diagram of co-cited authors created using VOSviewer, revealing strong co-occurring links between the top 100 co-cited authors with the highest number of citations. Notably, highly productive authors include Trosko, Je, Laird, DW, and Yamasaki, H. **Table 3** lists the top ten authors and co-cited authors with significant contributions in the field. It is evident that NAUS, CCG is the most prolific author in terms of article output, with 37 papers published in the last 24 years, followed by Laird, DW (n=27) and Mesnil, Marc (n=20). The three most-cited authors are NAUS, CCG (n=1,963), Laird, DW (n=1,742), and Lampe, PD (n=2,314), with Lampe, PD’s article having the highest number of citations (n=2,314) and the highest average citation rate (n=144.63). These authors are leaders in the field of research. The H-index, which combines productivity and impact, is a comprehensive measure of researchers’ contributions to the field (32). As shown in **Figure 5B**, it is clear that the most-cited authors, NAUS, CCG and Laird, DW, also have the highest H-index scores. The H-index is positively correlated with the citation dominance of more frequently cited and widely recognized authors.

**Table 3:** The top 10 most prolific and cited authors.

Rank	Authors	Documents	Citations	Average citations	Total link strength	Co-cited authors	Citations	Total link strength
1	Naus, CCG	37	1963	53.05	47	Trosko, Je	466	6987
2	Laird, DW	27	1742	64.52	39	Laird, DW	459	8791
3	Mesnil, Marc	20	631	31.55	43	Yamasaki, H	395	6412
4	Trosko, JE	17	1083	63.71	18	Mesnil, M	387	6469
5	Lampe, PD	16	2314	144.63	19	Naus, CCG	320	6015
6	Leithe, Edward	15	622	41.47	45	Loewenstein, Wr	295	5593
7	Bertram, JS	14	914	65.29	6	Huang, RP	284	4711
8	Czyz, Jaroslaw	14	282	20.14	32	Lampe, PD	279	5421
9	Madeja, Zbigniew	14	293	20.93	30	Solan, JL	247	4807
10	Brehm, Ralph	13	339	26.08	5	Leithe, E	243	5395

### 3.4 Visualization and analysis of journals and co-cited journals

A total of 612 journals published articles related to Cx43 in solid tumors from 2000 to 2024. **Table 4** lists the top 10 journals by the number of publications in this research area, as well as the top 10 journals by the number of co-cited publications. Web of Science reported the Journal Citation Report (JCR) and Impact Factor (IF) for the year 2023. The IF is a metric primarily used to evaluate the quality, importance, and impact of journals (31). In the top 10 ranking by the number of articles published, only one journal had an IF value greater than 10. Seven journals were classified in the Q1 JCR category, and two journals were classified in the Q2 JCR category. The International Journal of Molecular Sciences published the highest number of articles (n=43, IF:5.6). The journal with the highest impact factor (IF:11.7), Cancer Research, had the highest total citations and average citations (Citations: 3,449, Average citations: 149.96), despite publishing only 23 papers. In terms of co-citations, J Biol Chem leads with 2,598 citations. This journal generally publishes research related to biochemistry and molecular biology. Cancer Research follows closely with 2,596 citations and focuses primarily on basic and clinical cancer research. Other journals with significant citation numbers include Proc Natl Acad Sci USA (n=1,862), Carcinogenesis (n=1,763), and J Cell Biol (n=1,578).

**Table 4:** The top 10 issued and cited journals for Cx43 in solid tumor.

Rank	Journal	documents	citations	Average citations	JCR	IF	Co-cited Journal	citations	JCR	IF
1	International Journal of Molecular Sciences	43	601	13.98	1	5.6	Journal of Biological Chemistry	2598	2	4.4
2	Plos One	38	1223	32.18	1	3.3	Cancer Research	2596	1	11.7
3	Journal of Biological Chemistry	28	2235	79.82	2	4.4	PNAS	1862	1	10.8
4	Cancers	24	314	13.08	1	4.9	Carcinogenesis	1763	2	3.7
5	Carcinogenesis	24	1245	51.88	2	3.7	Journal of Cell Biology	1578	1	8
6	Scientific Reports	24	474	19.75	1	4.3	Nature	1212	1	54.4
7	Cancer Research	23	3449	149.96	1	11.7	Journal of Cell Science	1176	3	3.9
8	Cancer Letters	21	608	28.95	1	8.3	Cell	1063	1	66.85
9	International Journal of Oncology	19	520	27.37	1	4.6	Oncogene	1056	1	7.5
10	Molecular Carcinogenesis	18	411	22.83	3	3.8	BBA-Biomembranes	1042	3	3.3

**Figure 6:**
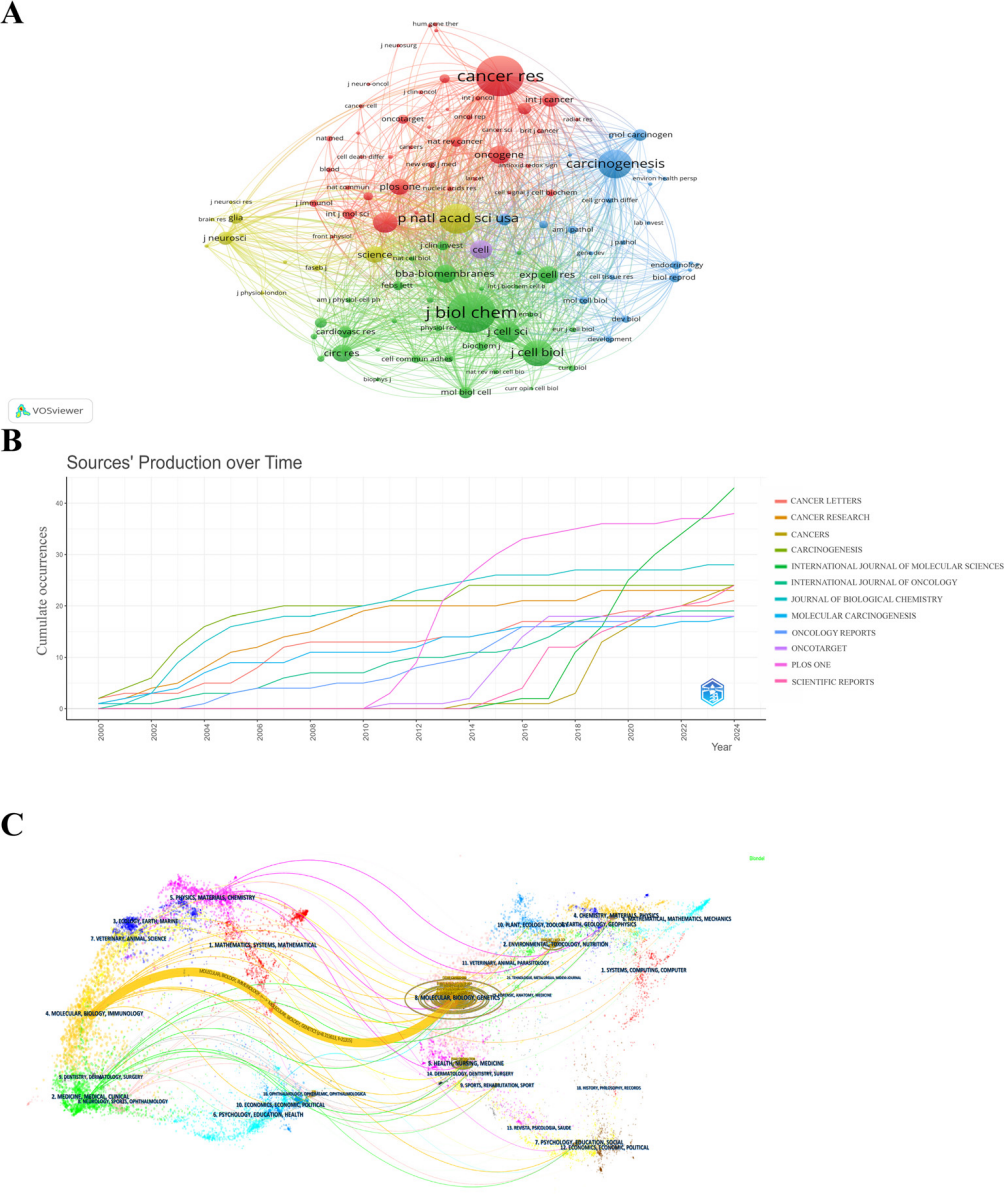
**(A)** Network diagram of journals for Cx43 studies. **(B)** Source production over time. **(C)** Dual-map of journals from citing documents to cited references.

**Figure 6A** is a network diagram created by VOSviewer, where different colors represent different clusters of co-cited journals. The size of each circle corresponds to the number of published articles, and the number of lines and the distance between two circles indicate the degree of connectivity. The most prominent journal in terms of co-citations is the Journal of Biological Chemistry, followed by Cancer Research, as shown in **Figure 6A**. **Figure 6B** shows that Cancer Letters, Cancer Research, and Cancer have made significant contributions to the field from 2000 to 2024. In terms of publication output, PLoS One saw an increase in the number of articles starting in 2010, while Carcinogenesis began to rise in 2016, quickly surpassing its peers and maintaining a leading position through 2024. **Figure 6C** illustrates a double mapping of journals from cited literature to cited references, visualizing the research progress and frontiers in the field (33). The orange line represents articles related to molecular biology and genetics, while the main cited articles are associated with molecular biology and immunology. This indicates that publications in the fields of molecular biology and immunology (yellow track) have been significantly influenced by molecular biology and genetics (z=8.31, f=21,315).

### 3.5 Analysis of co-cited references

**Table 5** lists the top 10 most-cited publications in the field of Cx43. The study by Ponti D et al., titled “Isolation and *in vitro* propagation of tumorigenic breast cancer cells with stem/progenitor cell properties,” is the most cited (n=1,469). It was published in Cancer Research, one of the leading journals in the field of cancer research, which is widely recognized as an authoritative platform for both basic and clinical cancer-related studies. The second most-cited article is by Chen Q, published in Nature. The third most-cited article is a review by Paul DL et al. on the effect of Cx43 phosphorylation on gap junction communication.

**Table 5:** The top 10 most cited articles.

Title	Paper	DOI	First Author	Year	Journal	Total Citations
Isolation and *in vitro* propagation of tumorigenic breast cancer cells with stem/progenitor cell properties	PONTI D, 2005, CANCER RES	10.1158/0008-5472.CAN-05-0626	Dario Ponti	2005	Cancer Research	1469
Carcinoma-astrocyte gap junctions promote brain metastasis by cGAMP transfer	CHEN Q, 2016, NATURE	10.1038/nature18268	Qing Chen	2016	Nature	663
The effects of connexin phosphorylation on gap junctional communication	LAMPE PD, 2004, INT J BIOCHEM CELL B	10.1016/S1357-2725(03)00264-4	Paul D Lampe	2004	Int J Biochem Cell Biol	486
Connexin43 phosphorylation: structural changes and biological effects	SOLAN JL, 2009, BIOCHEM J	10.1042/BJ20082319	Joell L Solan	2009	Biochemical Journal	464
Phosphorylation of connexin43 on serine368 by protein kinase C regulates gap junctional communication	LAMPE PD, 2000, J CELL BIOL	10.1083/jcb.149.7.1503	P D Lampe	2000	Journal of Cell Biology	461
Cx43 hemichannels and gap junction channels in astrocytes are regulated oppositely by proinflammatory cytokines released from activated microglia	RETAMAL MA, 2007, J NEUROSCI	10.1523/JNEUROSCI.2042-07.2007	Mauricio A Retamal	2007	Journal of Neuroscience	399
Overview of mechanisms of action of lycopene	HEBER D, 2002, EXP BIOL MED	10.1177/153537020222701013	David Heber	2002	Experimental Biology and Medicine (Maywood)	361
Mechanisms of cardiac and renal dysfunction in patients dying of sepsis	TAKASU O, 2013, AM J RESP CRIT CARE	10.1164/rccm.201211-1983OC	Osamu Takasu	2013	Am J Respir Crit Care Med	349
Heterogeneity for stem cell-related markers according to tumor subtype and histologic stage in breast cancer	PARK SY, 2010, CLIN CANCER RES	10.1158/1078-0432.CCR-09-1532	So Yeon Park	2010	Clinical Cancer Research	317
Concentration-dependent inhibition of angiogenesis by mesenchymal stem cells	OTSU K, 2009, BLOOD	10.1182/blood-2008-09-176198	Keishi Otsu	2009	Blood	278

**Figure 7:**
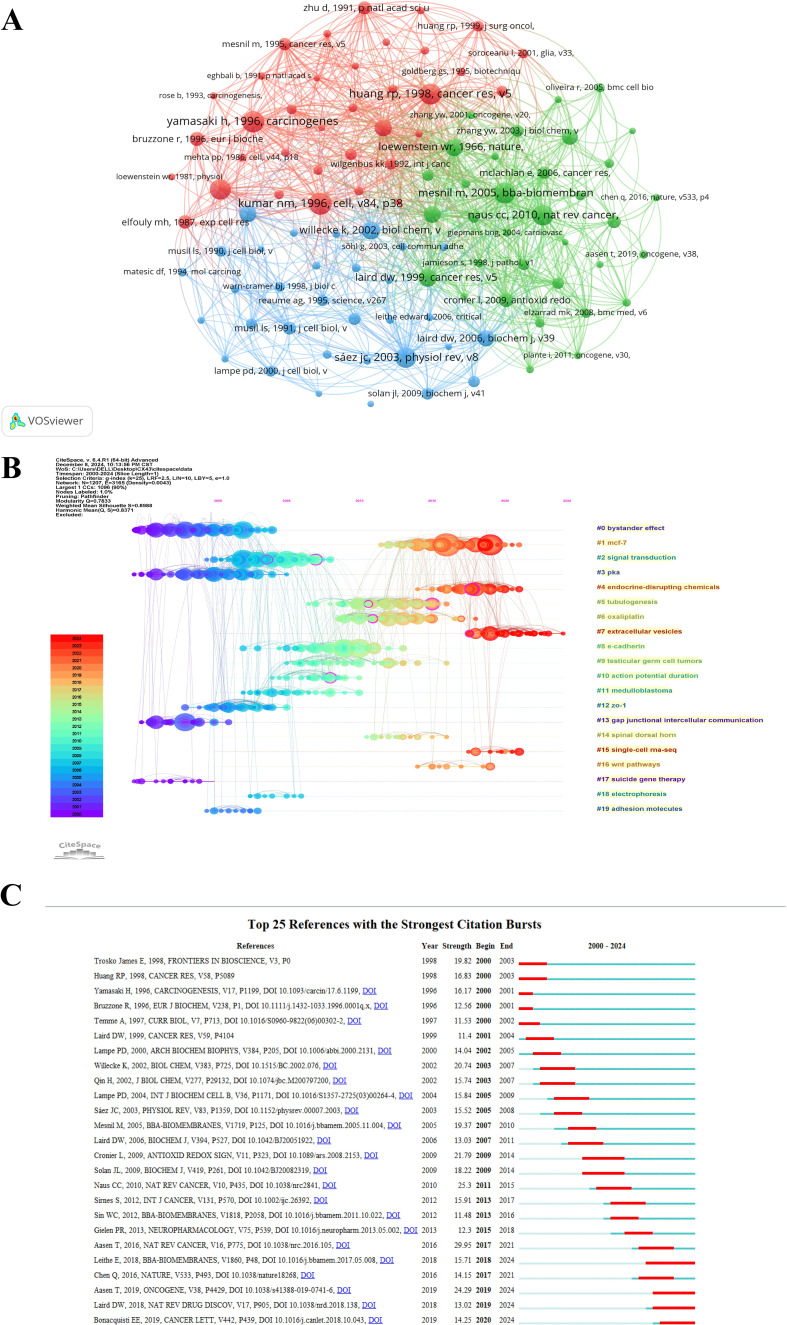
Co-citation analysis of cited references. **(A)** Network map of co-cited references. **(B)** Timeline plot of literature co-citations clustering. **(C)** The top 25 references with the strongest citation bursts.

The research progress of Cx43 in solid tumors was illustrated through literature co-citation analysis. **Figure 7A** presents the co-citation network, obtained by clustering the cited literature into 20 clusters. The cluster module value (Q) is 0.7833, and the mean cluster contour value (S) is 0.8988. Since S > 0.7, this indicates that the clusters have a significant structure and a convincing clustering effect. Additionally, **Figure 7B** shows a timeline view of co-cited references from 2000 to 2024. This timeline provides a clearer picture of historical findings and the intrinsic connections within each cluster. In the figure, nodes placed along the same line represent a cluster, with the themes of the clusters labeled using abstract terms from the cited articles. The larger the node, the higher the co-citation frequency. Two nodes connected by a curve indicate simultaneous citation by another article. The color of the citation ring represents the temporal distribution of citations. In the study of Cx43, early research focused on the bystander effect, PKA signaling pathways, and GJIC function. In recent years, research has gradually shifted to emerging themes such as “#1 MCF-7,” “#4 endocrine-disrupting chemicals,” “#7 extracellular vesicles,” and “#15 single-cell RNA-seq”.

The outbreak analysis of the literature aims to identify works that have experienced a significant increase in citation frequency over a specific time period, helping to uncover hotspots and trends in Cx43 research in solid tumors. To visualize this, **Figure 7C** was created. The green line in the figure represents the years 2000-2024, while the red line indicates the duration of the outbreak. Notably, the article “Gap junctions and cancer: communicating for 50 years” (2017-2021, intensity: 29.95) by Aasen T, published in 2016, exhibits the highest outbreak intensity. Additionally, a scholarly work that showed high intensity levels in the early years is “Structural and functional diversity of connexin genes in the mouse and human genome” by Willecke K (2003-2007, intensity: 20.95). A more recent high-intensity work is “Connexins in cancer: bridging the gap to the clinic” by Aasen T (2019-2024, intensity: 24.29). This article reviews the current role of connexins and gap junctions in cancer, with a particular focus on recent advances in their prognostic and therapeutic potential.

### 3.6 Keyword co-occurrence, clustering and bursting

**Figure 8:**
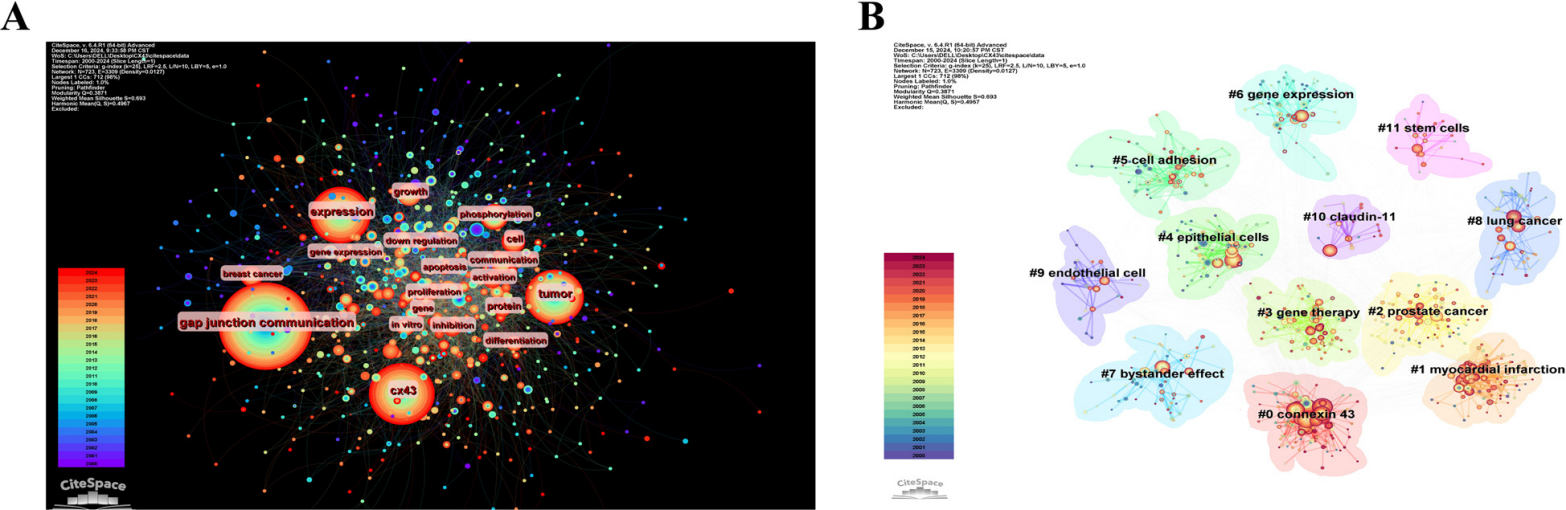
Visualization of keywords analysis. **(A)** The network map of keywords. **(B)**) Clusters of keywords.

**Figure 8A** shows the statistics for high-frequency keywords in the included literature. As expected, “gap junction communication,” “Cx43,” and “tumor” ranked high, while other common keywords such as “expression,” “growth,” “cell,” and “breast cancer” also appeared more than 150 times, reflecting the main research focus in this area. Keyword clustering illustrates the frequency and distribution of two keywords appearing together, forming clusters that represent the main research directions in the field, as shown in **Figure 8B**. These clusters include “connexin 43,” “myocardial infarction,” “prostate cancer,” “gene therapy,” “epithelial cells,” “cell adhesion,” “gene expression,” “bystander effect,” “lung cancer,” “endothelial cells,” “claudin-11,” and “stem cells”.

**Figure 9:**
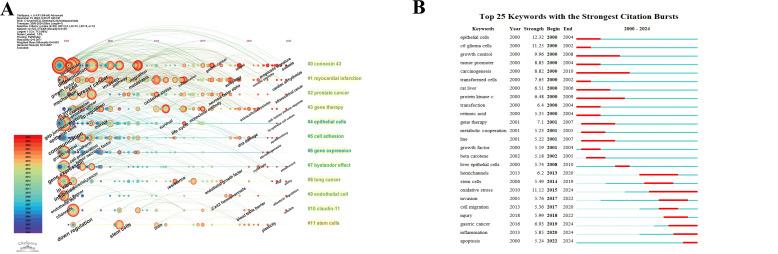
**(A)** Keyword timeline view of Cx43 in solid tumor research. **(B)** Top 25 keywords with the strongest citation bursts.

Based on these 12 clusters, a cluster timeline was created, as shown in **Figure 9A**. Each cluster is represented by a horizontal line, with the same color indicating the same cluster. The horizontal line for each cluster progresses from left to right, representing the development of the keywords over time. The node color changes from blue to red, indicating when the keywords appeared, with blue representing the earlier years and red representing areas of research that have gained prominence in recent years. **Figure 9A** depicts the chronological order of the grouped keywords, providing a comprehensive visualization of the evolution and trends of the research clusters. Additionally, we removed the keywords “gap junction communication,” “Cx43,” “tumor,” and “expression” from the list of high-frequency keywords. From the perspective of the temporal development of keywords, the representative keywords in recent years include: “breast cancer,” “prognostic signatures,” “gastric cancer,” “tumor microenvironment,” “anti-tumor immune response,” “target,” “phosphorylation,” “metastasis,” and “resistance,” highlighting the research hotspots in recent years.

Keyword burst analysis using CiteSpace provides insights into the prevalence and temporal distribution of research in this area, helping to predict future trends. **Figure 9B** presents the 25 keywords with the most significant bursts of interest, categorized into four main groups: Cell Proliferation and Migration, Biochemistry and Molecular Biology, Cancer Therapeutics and Clinical Research, and Cancer Biological Processes and Mechanisms. The keyword “epithelial cells” received the earliest attention and had the highest burst intensity, with an intensity of 12.32 for the period 2000-2004. This suggests that research in the early years mainly focused on the proliferation and migration of cancer cells. The keyword bursts in the period 2015–2024 include “oxidative stress,” “gastric cancer,” “inflammation,” and “apoptosis,” reflecting that altered Cx43 expression may impact tumor growth and drug resistance by regulating oxidative stress, pro-inflammatory factor release, and apoptosis. These keywords indicate that changes in Cx43 expression can influence tumor growth and drug resistance by regulating oxidative stress, inflammatory responses, and apoptosis. Additionally, Cx43 may serve as an important molecular marker and a potential therapeutic target for tumor treatment. This not only deepens our understanding of tumor biology but also lays the groundwork for personalized medical treatments.

## 4 Discussion

### 4.1 Relevance to general information on publications

Research on Cx43 began to focus on its role in tumors as early as 1990. Initial studies highlighted the importance of Cx43, a cardiac gap junction protein, in communication between tumor cells. M. Oyamada et al. demonstrated that Cx43 expression was elevated in hepatocellular carcinoma, while its expression in surrounding non-tumor liver tissue was extremely low (34). Subsequent studies have shown that alterations in Cx43 expression are closely associated with tumorigenesis, progression, and metastasis (35–37). Cx43 dysfunction has been observed in a variety of tumor types, including bladder cancer (38), esophageal cancer (39), and breast cancer (40), where it disrupts intercellular communication. Although our study focuses on literature published after 2000, these early findings from the 1990s provided a critical foundation for subsequent research on Cx43.

To our knowledge, this study is the first bibliometric analysis of Cx43 in solid tumors. We searched the WoSCC database for 1,666 publications on Cx43 and solid tumors from 61 countries, 612 journals, 1,706 institutions, and 8,563 authors, covering the period from 2000 to 2024. The results of the study show a steady growth trend in both the number of published papers and citations. Notably, the number of publications peaked in 2020 with a total of 98, a surge closely linked to significant advancements in Cx43 research, particularly in the areas of breast cancer and glioma in recent years. Among the top 10 most productive countries in this field, the United States and China have published a substantial number of articles. In terms of citation-weighted productivity, Italy and Canada lead other countries. Although their publication volumes are relatively low, their articles are cited more frequently. In contrast, China has a high research output, but the citation impact of its articles is relatively lower. This may be due to factors such as research quality, limited international influence, and the fact that many of the papers are published in domestic rather than international journals. The United States ranks first in both publication volume and citation counts, and also has a high citation-weighted productivity. The U.S. maintains its leadership due to its strong research infrastructure, ample funding support, and technological innovation, consistently driving Cx43 cancer research forward (8, 13, 41). China’s high output is largely attributed to the increasing cancer incidence rates, particularly for cancers like lung, gastric, liver, and breast cancer. Additionally, China’s rapidly developing biopharmaceutical industry and vast clinical sample banks have made it an increasingly influential force in oncology research (42–45). Among the top ten institutions by publication volume, two Canadian institutions stand out, underscoring Canada’s dominant position and significant influence in Cx43 and solid tumor research. Notably, the University of Western Ontario in Canada is the leading institution in this field, with its Cancer Research Centre and Cell Communication Laboratory focusing on Cx43-related research (46–48). However, despite China’s strong competition in terms of publication volume, its average citation count remains lower, suggesting that the quality of research in China lags behind that of other high-output countries. When it comes to researchers, the most influential author is Christian C.G. Naus from the University of Western Ontario, who further solidifies Canada’s leadership in this area. Naus’ 2010 review article in Nature Reviews Cancer extensively discussed the relationship between connexins and cancer, highlighting Cx43’s tumor-suppressive role in human solid tissue cancers and its potential as a conditional tumor suppressor that regulates tumor cell proliferation, adhesion, and migration (17). Naus’ team has focused on exploring the role of gap junctions in neurodevelopment and diseases, making significant advances in cell biology and cancer research, particularly in the reconstruction of gap junction communication, where their engineered tumor cells show inhibitory effects on growth and tumorigenesis (49–51). Regarding journal publications, the top ten journals account for 15.73% of total publications, reflecting their academic influence in the field. Notably, the Journal of Biological Chemistry and Cancer Research have lower impact factors compared to some journals but are cited far more frequently. Among co-cited journals, Cell has the highest impact factor. Known for publishing high-quality articles, Cell caters to researchers across life sciences, with a special focus on cell biology, molecular biology, cancer research, and immunology (52, 53). A journal overlay visualization analysis shows that early research primarily focused on molecular mechanisms. With the development of immunosuppressive therapies, Cx43 expression has had a significant impact on tumor cells’ response to immune checkpoint inhibitors. Several studies have explored Cx43’s potential in tumor immunology (46). Therefore, Cx43’s importance in solid tumors has garnered widespread attention, particularly in its roles in tumorigenesis, progression, metastasis, and response to immunotherapy.

### 4.2 Advancements and promising directions

References provide a solid foundation and developmental trajectory for research in the field, serving as the cornerstone for building a body of knowledge. By analyzing the relevant literature and extracting keywords, we are able to capture a wealth of knowledge in this cutting-edge field and gain insights into current research hotspots and future trends. Over the past two decades, Cx43 has gradually become a significant research focus. Popular terms associated with Cx43 include “phosphorylation,” “breast cancer,” “gastric cancer,” “target,” “anti-tumor immune response,” “tumor microenvironment,” “prognostic signature,” and “resistance.” In these studies, Cx43 has garnered considerable attention. It has been shown that the phosphorylation of Cx43 plays a crucial role in tumorigenesis, progression, and metastasis, particularly in tumor cell migration and invasion (54). Cx43 has also been proposed as a prognostic biomarker for solid tumors, potentially aiding in early diagnosis and prognosis prediction (7, 55). Furthermore, Cx43 is considered a potential target for immunotherapy, with its modulation possibly improving tumor immune escape mechanisms and enhancing anti-tumor immune responses (56). Additionally, Cx43’s role in tumor drug resistance has drawn significant attention. Investigating its involvement in the mechanism of drug resistance may offer new directions for overcoming this challenge (57, 58). Thus, Cx43, as a key target for tumor therapy and prognostic assessment, provides valuable insights for future research and clinical applications. The following section further discusses these points.

#### 4.2.1 Role and mechanism of Cx43 phosphorylation in cancer progression

Based on the co-cited references analysis in section 3.5, the third, fourth, and fifth most-cited papers focus on the mechanism of Cx43 phosphorylation. This section examines how Cx43 phosphorylation is regulated in cancer, with examples from gastric cancer, breast cancer, lung cancer, and glioma.

Recent studies have shown that the phosphorylation of Cx43 exhibits a significant tissue-specific regulatory pattern in the initiation and progression of different types of tumors. The expression of phosphorylated Cx43 (P-Cx43) was notably upregulated in breast hyperplasia, fibroadenoma, and breast cancer. In particular, in carcinoma *in situ* and invasive carcinoma, P-Cx43 is strongly expressed not only in residual mammary epithelial cells but also widely distributed in transformed luminal cells and capillary endothelium within and adjacent to the tumor (59). Cx43 is also strongly associated with lung cancer (60). In normal lung tissue, Cx43 is in a hypophosphorylated state, but in lung tumors, phosphorylation levels are significantly increased (61). This increase may be due to Cx43 promoting the proliferation, migration, and angiogenic capacity of lung microvascular endothelial cells by regulating phosphorylation at its C-terminal Ser279 site (62). In high-grade gliomas, Cx43 expression is significantly downregulated, while P-Cx43 expression is markedly upregulated, suggesting that the phosphorylation status of Cx43 may be closely linked to tumor malignancy (63). Overall, these differential expression patterns suggest that Cx43 phosphorylation may play a role in regulating the malignant progression of various tumor types through distinct molecular mechanisms.

Firstly, the phosphorylation of Cx43 affects the permeability, conductivity, and gating properties of GJIC, which, in turn, impacts intercellular communication and influences physiological or pathological processes both *in vitro* and *in vivo* (64). For example, as early as 1998, researchers proposed that the phosphorylation of Cx43 in breast cancer tumor cells is associated with interactions between tumor cells and endothelial cells. These interactions significantly increase the level of tyrosine phosphorylation of Cx43, which in turn inhibits GJIC in endothelial cells (65). The transient loss of GJIC may promote the extravasation of tumor cells from the bloodstream, a critical step in metastasis.

Secondly, Phosphorylation of Cx43 typically occurs in its intracellular C-terminal region, and various kinases regulate its function through phosphorylation. Common phosphorylation sites include serine (Ser) and threonine (Thr) residues. Different kinases, such as protein kinase A (PKA), protein kinase C (PKC), and mitogen-activated protein kinase (MAPK), can regulate the function of Cx43 by phosphorylation (66, 67). In gastric cancer and lung adenocarcinoma (LUAD), key components of the PI3K/AKT/mTOR signaling pathway, such as AKT, are frequently activated by aberrant phosphorylation. In particular, hyperphosphorylation of AKT is closely associated with the malignant phenotype of tumors (68–71). In the PI3K/AKT signaling pathway, AKT promotes Cx43 function by phosphorylating the Ser373 site of the Cx43 hemichannel, which facilitates hemichannel opening and ATP release, driving cell migration and reactive changes (72). This abnormal activation of AKT promotes tumor cell proliferation, survival, and invasion. In gliomas, the viability and migratory capacity of glioma cells are significantly reduced after treatment with PKC, MAPK, and PTK inhibitors (63). Taken together, phosphorylation of Cx43 regulates its function through different kinase pathways in a variety of cancer types, promoting tumor cell proliferation, migration, and invasion, making it a key regulatory mechanism in cancer development and progression.

In addition, the phosphorylation status of Cx43 and its interaction with related kinases can regulate the activity of GJIC, which in turn affects tumor cell proliferation, migration, and invasion (73). For example, oxidized resveratrol (ORes) significantly inhibited the proliferation and growth of breast cancer cells by inducing ferroptosis through inhibition of the EGFR/PI3K/AKT/GPX4 signaling axis (74). The phosphorylation status of Cx43 in glioblastoma multiforme (GBM) may affect the activity of the downstream signaling pathway of EGFR by modulating its binding ability to Akt/ERK (75). In contrast, EGFR can mediate the protein kinase C (PKC) signaling pathway, which rapidly induces phosphorylation of Cx43 at the Ser368 site, leading to a decrease in GJIC activity (76). Taken together, EGFR may play a role in the progression of breast cancer and glioma by affecting intercellular communication through Cx43 phosphorylation. Notably, Cx43 mutations that block MAPK phosphorylation sites significantly reduced Cx43 hemichannel activity and improved pathological outcomes in a stroke model, further supporting the importance of Cx43 phosphorylation in disease progression (49). The mechanism may be that when Cx43 at the MAPK phosphorylation site is mutated, the openness of Cx43 hemichannel is inhibited, leading to impaired GJIC.

In summary, phosphorylation of Cx43 plays an important biological role in different types of cancers by regulating its function and GJIC activity. Changes in the phosphorylation status of Cx43 are closely associated with tumorigenesis, progression, and malignant phenotypes. Different kinase pathways, such as PI3K/AKT, MAPK, and others, are involved in this process. Through phosphorylation, Cx43 affects key processes such as intercellular communication, cell proliferation, migration, and invasion, which in turn drive tumor growth and metastasis. Meanwhile, the tissue-specific expression of Cx43 phosphorylation in different cancers indicates its diversity and complexity in tumor progression. These studies provide new perspectives and further reveal the critical regulatory role of Cx43 phosphorylation in tumor cell behavior and cancer progression, as well as potential targets for cancer therapy.

#### 4.2.2 Prognostic biomarkers for solid tumors

Biomarkers can indicate normal or abnormal processes and are useful for diagnosis, prognostic assessment, and treatment monitoring of diseases. Among the connexins studied so far, only Cx43 has been associated with improved disease prognosis and serves as a better prognostic marker than vascular infiltration or necrosis, commonly used as markers in solid tumors such as breast cancer, gastric cancer, glioma, and lung cancer (7, 55, 77–81). In this section, in conjunction with the 3.6 Results section, we expand on the research progress of Cx43 as a diagnostic marker for breast and gastric cancer. First, high levels of Cx43 may promote tumor progression in biopsies from patients with advanced breast cancer (82). Wu et al. found that Cx43 deficiency was significantly associated with poor recurrence-free survival in patients treated with tamoxifen (TAM), and its expression level was closely linked to patient prognosis (83, 84). This suggests that Cx43 could serve as both a prognostic marker and a therapeutic target. Mechanistically, Cx43 deficiency leads to the loss of breast epithelial cell polarity, which is a marker of tumor initiation. Additionally, Cx43 regulates tumor progression through interactions with non-coding RNAs such as circRNAs and miRNAs (85). These dysregulated non-coding RNAs are highly stable in the circulatory system and not only serve as liquid biopsy markers (86), but also offer a new strategy for the early detection and prevention of breast cancer through the Cx43-mRNA-circRNAs-miRNAs regulatory axis (81).

Secondly, in gastric cancer, low expression of Cx43 was significantly associated with poorer cancer-specific survival (CSS) in patients following gastrectomy, and combined pathological stage and differentiation type were identified as independent predictors of CSS (87), suggesting that Cx43 could serve as a prognostic marker. Further studies have shown that H. pylori infection can promote gastric carcinogenesis by down-regulating Cx43 expression. A comparison of gastric cancer, precancerous lesions, and chronic gastritis tissue samples confirmed that Cx43 expression is negatively correlated with H. pylori infection (88). These findings suggest that Cx43 has important clinical implications for prognostic assessment and molecular diagnosis in gastric cancer.

#### 4.2.3 Dual role of Cx43 in immunotherapy: synergy and resistance

Cx43 is a key gap junction protein in the immune system, and it has been shown to not only mediate direct GJIC by specifically localizing at the cytotoxic T lymphocytes (CTLs) immunological synapse to form functional gap junctions (47), but also to influence chemokine secretion and complement pathway activation by modulating the interaction between macrophages and parenchymal cells, significantly impacting immune system function (89). Therefore, this study explores the role of Cx43 in immunotherapeutic resistance and its potential for synergistic therapeutic effects through both direct cell-to-cell communication and by influencing chemokine secretion and complement pathway activation.

First, Cx43 plays an important immune-promoting role in immunotherapeutic synergy through GJIC. It facilitates communication between immune cells such as T cells, B cells, and antigen-presenting cells, thereby enhancing the immune system’s ability to recognize and eliminate tumor cells (16, 90). Researchers have developed a Cx43-M2 activating antibody, which significantly increased the number and function of tumor-infiltrating effector T cells while reducing immunosuppressive regulatory T cells, resulting in a substantial enhancement of the anti-tumor immune response (91–93). Granzyme, a protease released by activated immune cells, plays a crucial role in target cell killing (94, 95). It has been shown that Cx43 can indirectly participate in immunotherapy by facilitating the transmembrane transport of effector molecules such as granzyme B. Blockade of Cx43 inhibits granzyme B activity (96). Cyd Soriano et al. discovered that the granzyme B inhibitor PI-9 was highly expressed in lung cancer cell lines, and an increase in PI-9 expression was also observed in primary cancer cells, inhibiting granzyme B-mediated cellular toxicity and enabling immune escape (97). In summary, we propose that Cx43 may mediate cytotoxicity in immunotherapy by promoting the release of granzyme, thereby enhancing tumor recognition and clearance by immune cells.

In 2020, the Society for Immunotherapy of Cancer (SITC) highlighted the resistance of advanced tumors to treatment with immune checkpoint inhibitors (ICIs) (98). Identifying the mechanisms behind tumor immune resistance and screening for populations that would benefit from treatment remain pressing challenges in modern medicine. The role of Cx43 in immunotherapy resistance has garnered increasing attention from researchers. In a Lewis lung cancer (LLC) model, knockdown of Cx43 results in reduced T-cell activation, making the tumor less responsive to anti-PD-1 therapy and further exacerbating immunotherapy resistance (46). GJIC is also an essential mechanism; for example, in hypoxic melanoma cells, Cx43-mediated gap junctions facilitate the transfer of hypoxia-induced miR-192-5p, significantly inhibiting the activity of CTLs and thereby aiding tumor immune escape (56). Moreover, due to its critical role in communication between dendritic cells and T cells, Cx43 mediates antigen transfer between dendritic cells (DCs), which further exacerbates immunotherapeutic resistance (99). Anastasia’s research demonstrated that in Keap1-mutated lung adenocarcinomas, the dendritic cell-T-cell response is significantly inhibited, driving immunotherapy resistance. However, a combination of glutaminase inhibition and immune checkpoint blockade reversed this immunosuppression, restored dendritic cell and T-cell responses, and made Keap1 mutant tumors more sensitive to immunotherapy (100). These findings highlight the crucial role of Cx43 in immunotherapy resistance, particularly its regulation of immune messaging between dendritic cells and T cells. Targeting Cx43 channels may offer a novel therapeutic strategy for overcoming immune resistance, and combining immune checkpoint inhibition with Cx43-mediated gap junction regulation could improve tumor response to immunotherapy and enhance anti-tumor immune responses.

The dual role of Cx43 in both immunotherapeutic synergy and immune resistance positions it as a potent therapeutic target, while also potentially serving as a barrier in tumor immunotherapy. Its ultimate effect is highly dependent on factors such as tumor type, microenvironmental conditions, and immune status (101). Therefore, precise regulatory strategies targeting Cx43 must be tailored to specific tumor types and microenvironmental conditions, with optimal immunotherapeutic efficacy achievable only through differentiated intervention protocols. This understanding provides a crucial theoretical foundation and practical guidance for the development of novel tumor immunotherapy strategies based on Cx43 regulation.

#### 4.2.4 Multidimensional regulatory mechanisms of Cx43 in drug resistance in solid tumors

Cx43 plays a pivotal role in regulating drug resistance in solid tumors, with its mechanisms showing significant tissue-specific and treatment-specific dependence. The regulation of treatment response by Cx43 occurs through multiple dimensions, including changes in expression levels, interaction with signaling pathways, and gap junction functionality. Changes in Cx43 expression levels represent one of the core mechanisms regulating drug resistance, but its impact on treatment response varies depending on the cancer type. For instance, in glioma, both the ectopic expression and upregulation of Cx43 lead to resistance to temozolomide (TMZ) (57, 102–104). In pancreatic cancer, the loss of Cx43 expression promotes chemotherapy resistance (105), whereas in non-small cell lung cancer (NSCLC), low expression of Cx43 results in cisplatin resistance, which can be reversed by Artemisinin B (Art B) through upregulation of Cx43 (19). These studies indicate that Cx43 expression plays a key role in chemotherapy resistance across various cancers, and modulating Cx43 may help overcome this resistance.

In addition to directly regulating drug sensitivity, Cx43 also modulates resistance indirectly by influencing several signaling pathways. For example, in lung cancer cells, the inhibition of Cx43 induces macrophage polarization toward the pro-tumor M2 phenotype (TAM) and negatively regulates the cGAS-STING pathway in macrophages, leading to resistance to immune checkpoint inhibitors (46). In hormone-sensitive breast cancer, tamoxifen resistance is closely related to epithelial-mesenchymal transition (EMT), which is regulated by the c-Src/PI3K/Akt pathway (84). Interestingly, in glioma, Gui et al. further demonstrated that upregulation of Cx43 promotes β-catenin membrane retention, inhibits TCF/LEF transcriptional activity, and disrupts miR-205-5p expression, ultimately activating the E2F1/ERCC1 axis and triggering chemotherapy resistance (58). These studies highlight the complex mechanisms by which Cx43 regulates resistance through the modulation of multiple signaling pathways.

The gap junction functionality of Cx43 also plays a crucial role in drug resistance. For example, in HER2-positive breast cancer cells, the loss of Cx43 gap junction function is associated with resistance, while overexpressing Cx43 to restore gap junction activity can reverse this phenomenon (4). Additionally, photodynamic therapy (PDT) is widely used in early-stage cancer treatment, but the recurrence rate is high. Studies suggest that this may be related to Cx43 expression levels. Enhancing Cx43-mediated gap junction function increases the sensitivity of malignant cells to PDT, thereby improving treatment efficacy, whereas inhibiting gap junction function leads to PDT resistance (106). These findings suggest that the gap junction functionality of Cx43 plays a significant role in drug resistance, particularly in HER2-positive breast cancer and PDT treatment. Restoring its function could be a potential strategy to improve treatment sensitivity.

In conclusion, the role of Cx43 in drug resistance in solid tumors is complex and diverse, involving mechanisms such as expression regulation, signaling pathway modulation, and gap junction functionality. Although extensive research has revealed the involvement of Cx43 in various cancer types and treatment contexts, its precise mechanisms require further exploration. Future studies should focus on understanding the regulatory networks of Cx43, its role in the tumor microenvironment, and its potential as a therapeutic target to provide new strategies for overcoming solid tumor drug resistance.

#### 4.2.5 The challenges and prospects of translating Cx43 biology into therapeutic strategies

In 1978, Van R. Potter highlighted that cancer is not just a cellular issue, but rather a problem of cellular interactions (107). Connexins, especially Cx43, play a crucial role in the biology and pathology of cancer, extending beyond classical intercellular communication functions to include intracellular signaling, hemichannel activity, and exchanges with the extracellular environment (108). Due to its active involvement in cancer progression, Cx43 can serve as a biomarker for cancer (109) and as a potential drug target in cancer therapy (110). The combination of Cx43-targeted therapies with immunotherapy represents a promising direction in cancer treatment. Basic research shows that restoring Cx43 expression can significantly improve the immune synapse function between cancer cells, dendritic cells (DCs), and natural killer (NK) cells, promoting anti-tumor immune responses through enhanced GJIC (99). However, there are still significant challenges in this field. First, clinical translation research of Cx43 as a therapeutic target is severely lacking. Second, its mechanisms of action show significant disease stage, cancer type, and microenvironment specificity. These factors limit the development of clinical strategies based on Cx43 (111). Notably, recent studies offer breakthrough directions for addressing these challenges. Given the high specificity required for Cx43 regulation, current research has shifted toward developing pharmacological tools that precisely target specific functional domains of Cx43. In particular, significant progress has been made in the development of therapeutic peptides. These innovative peptides can enhance or inhibit hemichannel activity while preserving gap junction function, or specifically interfere with Cx43-mediated cellular signaling (112, 113). As these novel molecular tools continue to evolve, they could provide more precise treatment options for cancer and other diseases in the future, opening new avenues to improve patient outcomes and quality of life.

### 4.3 Limitations

One limitation of this study is that it only used the WoS database, without including other databases such as PubMed, Google Scholar, and Embase. This could lead to the omission of certain areas or research directions, especially in interdisciplinary studies. The main reason for this decision was that PubMed is specifically focused on biomedical and life sciences, whereas WoS covers a broader range of disciplines. While there is significant overlap between the two databases, not all articles indexed in PubMed are included in WoS. By focusing solely on WoS, we aimed to ensure that our analysis was comprehensive across a multidisciplinary research spectrum. Additionally, including databases like PubMed could bias the results toward biomedical topics, which might not reflect the broader scope we intended to study. Furthermore, we included only English-language literature, overlooking research conducted in other languages around the world. This decision was made because English is the primary language for international academic publishing and communication, and the majority of authoritative databases (such as PubMed and Web of Science) primarily feature English-language publications. To ensure the broad recognition and reproducibility of our research, we prioritized English-language, peer-reviewed literature. While research published in other languages is equally important, we relied mainly on English-language databases and existing systematic reviews during the literature selection process to minimize potential omissions. We also focused on literature published between 2000 and 2024. However, some recent publications from 2024 may not yet have been widely cited, which could affect their weight in the bibliometric analysis, potentially making certain emerging areas appear less significant. Despite these limitations, this study, through a systematic bibliometric analysis, aims to highlight the major trends and potential research hotspots in the study of Cx43 in solid tumors, providing important directions for future development in this field.

## 5 Conclusion

In our study, we visualized the progress of Cx43 research in solid tumors over the past 24 years through bibliometric analysis using CiteSpace, VOSviewer, and Bibliometrix to visualize the data and identify potential research directions. Research in this area continues to be active, with the number of publications increasing progressively each year. The United States stands out as the country with the highest output in Cx43 research related to solid tumors. Notably, there appears to be a gap between the number of publications and the impact or quality of research in China. Much of the research lacks sufficient innovation and depth to make a significant impact on the international academic community. The University of Western Ontario and CCG Naus are among the most influential institutions and researchers in the field. Based on the literature, we conclude that the research hotspots in Cx43 mainly focus on the role of Cx43 phosphorylation in tumorigenesis, progression, and metastasis, its potential as a prognostic biomarker for solid tumors, and Cx43 as a potential target for immunotherapy, as well as its key role in tumor drug resistance. These studies provide a comprehensive analysis for a deeper understanding of the role of Cx43 in solid tumors and help to promote further research in this area.

## Data Availability

The original contributions presented in the study are included in the article/supplementary material. Further inquiries can be directed to the corresponding author.
